# Gene expression and plant hormone levels in two contrasting rice genotypes responding to brown planthopper infestation

**DOI:** 10.1186/s12870-017-1005-7

**Published:** 2017-02-28

**Authors:** Changyan Li, Chao Luo, Zaihui Zhou, Rui Wang, Fei Ling, Langtao Xiao, Yongjun Lin, Hao Chen

**Affiliations:** 1 National Key Laboratory of Crop Genetic Improvement and National Centre of Plant Gene Research (Wuhan), Huazhong Agricultural University, Wuhan, 430070 China; 2 Hunan Provincial Key Laboratory of Phytohormones and Growth Development, Hunan Agricultural University, Changsha, 410128 China

**Keywords:** *Oryza sativa*, *Nilaparvata lugens*, cDNA microarray, Insect-resistance

## Abstract

**Background:**

The brown planthopper (BPH; *Nilaparvata lugens* Stål) is a destructive piercing-sucking insect pest of rice. The plant hormones salicylic acid (SA) and jasmonic acid (JA) play important roles in plant–pest interactions. Many isolated rice genes that modulate BPH resistance are involved in the metabolism or signaling pathways of SA, JA and ethylene. ‘Rathu Heenati’ (RH) is a rice cultivar with a high-level, broad-spectrum resistance to all BPH biotypes. Here, RH was used as the research material, while a BPH-susceptible rice cultivar ‘Taichung Native 1’ (TN1) was the control. A cDNA microarray analysis illuminated the resistance response at the genome level of RH under BPH infestation. The levels of SA and JA in RH and TN1 seedlings after BPH infestation were also determined.

**Results:**

The expression pattern clustering indicated that 1467 differential probe sets may be associated with constitutive resistance and 67 with the BPH infestation-responsive resistance of RH. A Venn diagram analysis revealed 192 RH-specific and BPH-inducible probe sets. Finally, 23 BPH resistance-related gene candidates were selected based on the expression pattern clustering and Venn diagram analysis. In RH, the SA content significantly increased and the JA content significantly decreased after BPH infestation, with the former occurring prior to the latter. In RH, the differential genes in the SA pathway were synthesis-related and were up-regulated after BPH infestation. The differential genes in the JA pathway were also up-regulated. They were jasmonate ZIM-domain transcription factors, which are important negative regulators of the JA pathway. Comparatively, genes involved in the ET pathway were less affected by a BPH infestation in RH. DNA sequence analysis revealed that most BPH infestation-inducible genes may be regulated by the genetic background in a trans-acting manner, instead of by their promoters.

**Conclusions:**

We profiled the analysis of the global gene expression in RH and TN1 under BPH infestation, together with changes in the SA and JA levels. SA plays a leading role in the resistance response of rice to BPH. Our results will aid in understanding the molecular basis of RH’s BPH resistance and facilitate the identification of new resistance-related genes for breeding BPH-resistant rice varieties.

**Electronic supplementary material:**

The online version of this article (doi:10.1186/s12870-017-1005-7) contains supplementary material, which is available to authorized users.

## Background

As a staple food for more than half of the world’s population, rice (*Oryza sativa*) is the most important crop in Asia [1], and insect pests are a main factor affecting rice production. Rice brown planthopper (BPH; *Nilaparvata lugens* Stål), which sucks sap from the plant’s phloem, is highly destructive [2]. In addition to direct damage, BPH, as the intermediate vector, can infect rice with pathogens, such as grass stunt virus and ragged stunt virus, leading to further yield losses [3, 4].

Since the 1960s, rice breeders have endeavored to identify BPH-resistant germplasms and develop BPH-resistant rice varieties. At least 30 BPH-resistance quantitative trait loci (QTLs) have been identified in rice. Among these, the chromosomal locations of 21 have been determined, and 12 have been fine-mapped. Most of these BPH-resistance genes were identified from wild rice, few from *indica* rice and none from *japonica* rice [5–7].

Four major BPH-resistance QTLs, *Bph14*, *Bph3*/*Bph17*, *BPH26*/*BPH2* and *Bph29*, were cloned using map-based techniques [8–11]. *Bph14* and *BPH26* encode coiled-coil, nucleotide-binding and leucine-rich repeat proteins that resemble the *R* genes of the nucleotide-binding–leucine-rich repeat family, which mediate plant resistance to pathogens [8, 10]. *Bph14* and *BPH26* are presumed to induce effector-triggered immunity, like the *R* genes, because they share similar conserved protein domains. The expression of *Bph14* is induced by BPH infestation, and it then activates the salicylic acid (SA) signaling pathway, induces calluses deposition in phloem cells and enhances trypsin inhibitor production [8]. *BPH26*, like *Bph14*, also mediates sucking inhibition in the phloem sieve element. The *Bph3* locus is a cluster of three genes encoding plasma membrane-localized lectin receptor kinases (*OsLecRK1*–*OsLecRK3*). *Bph3* was proposed to play a critical role in priming the pattern-triggered immunity response to BPH infestation by perceiving herbivore-associated or damage-associated molecular patterns due to the functions of recently discovered lectin receptor kinases [9]. *Bph29* is a recessive resistance gene that encodes a B3 DNA-binding domain that contains a protein that has a DNA mutation in the B3 domain. *Bph29* was proposed to have lost the function of the dominant allele, which is required for the settling of insects, and thus confers an antixenosis resistance in conjunction with an anther recessive locus [11]. The cloning of major BPH-resistance QTLs facilitated the understanding of the molecular mechanisms of resistant rice to BPH, although the biological mechanisms are not well understood.

In addition to the major BPH-resistance QTLs, many other rice genes that modulate BPH resistance (resistance-related genes), such as *OsHI-LOX* [12], *Bphi008a* [13], *OsERF3* [14], *OsPLD α4* and *α5* [15], *OsHPL3* [16], *OsACS2* [17], *OsAOC* [18], *Osr9-LOX1* [19], and *OsJMT1* [20], have recently been isolated. Most of them were identified using a reverse genetics strategy, and all of them are involved in the metabolism or signaling pathways of the plant hormones SA, jasmonic acid (JA) and ethylene (ET), indicating that these hormones play important roles in rice defense responses to BPH. SA is generally considered to positively regulate plant defense responses against pathogens and piercing-sucking insects, which cause minimal levels of physical injury to the host plants. The major BPH-resistance QTLs *Bph14* and *BPH29* enhance the expression of the SA synthesis-related genes and the homolog of Arabidopsis nonexpressor of pathogenesis-related genes 1, a key regulator of SA-dependent systemic acquired resistance after BPH infestation. They also suppress the expression levels of the JA synthesis-related genes and the ethylene signaling pathway receptor gene *ethylene insensitive 2* [8, 11].

JA is associated with wounding responses and positively regulates the plant defense response against chewing insects that cause extensive damage to the host. The interactions between SA and JA are commonly antagonistic, with SA having a suppression effect on JA accumulation and signaling. ET is considered to fine-tune the JA-induced responses, and JA and ET are synergistic. Many rice genes involved in JA metabolism, including *OsHI-LOX* [12], *OsPLDa4* and *-a5* [13], *OsHPL3* [16], *AOC* [18], *Osr9-LOX1* [19] and *OsJMT1* [20], can modulate BPH resistance. JA and its metabolites have diverse functions in BPH resistance. The ET pathway is generally considered to negatively modulate the BPH resistance of rice [21]. The ET-responsive gene *OsERF3* encodes a nucleus-localized protein, which is rapidly up-regulated in response to infestations of the rice striped stem borer (SSB; *Chilo suppressalis* Walker). Transgenic rice overexpressing *OsERF3* exhibit improved SSB resistance but are more susceptible to BPH. *OsACS2* encodes a rice 1-aminocyclopropane-1-carboxylic acid synthase gene, which is inducible by both SSB and BPH infestations. Suppression of *OsACS2* expression in rice reduced the elicited ethylene emissions and SSB resistance, but enhanced BPH resistance [17].

‘Rathu Heenati’ (RH), an *indica* rice cultivar from Sri Lanka, has a high, durable resistance to all of the BPH biotypes. RH is regularly used as a positive control when identifying BPH resistant rice varieties and is an important BPH-resistance donor for rice breeding. In this study, a cDNA microarray analysis was performed to profile the whole genome expression of RH, and a BPH-susceptible rice cultivar ‘Taichung Native 1’ (TN1) was used as the control. All of the rice plants were grown under three treatment conditions, natural growth (untreated), needle puncturing (mocking a simple mechanical wound) and BPH infestation, and two sampling time points, 6 and 24 h after treatment, were used. The changes in the levels of SA and JA in RH and TN1 seedlings were also determined at 6, 24 and 48 h after BPH infestation. The ET content is difficult to measure because of its volatility and was, therefore, not determined in this study. This study revealed the resistance responses of RH to BPH at the genomic transcription level and through changes in the JA and SA contents. The results of this study aid in understanding the biological foundation of the BPH resistance of RH and in identifying new BPH resistance-related genes for rice breeding.

## Results

### Genome-wide comparison of differentially-expressed probe sets between RH and TN1 under BPH infestation

The experimental design included two rice varieties (RH and TN1), three treatments (untreated control, needle puncturing and BPH infestation) and two sampling time points (6 and 24 h after BPH infestation). There were 12 samples in total, with 3 biological replicates per sample. The abbreviations R6C, R24C, R6N, R24N, R6P, R24P, T6C, T24C, T6N, T24N, T6P and T24P were used to represent these 12 samples, where the first letter, R or T, indicates the rice varieties RH or TN1, respectively; the Arabic number in the middle indicates the 6 or 24 h post-treatment collection time points; and the last letter C, N or P, indicates one of the three different treatments, untreated control, needle puncturing or BPH infestation, respectively. The Affymetrix GeneChip Rice Genome Array, containing 57,194 probe sets, was used for the microarray analysis, and the results of all 36 chips were submitted to the NCBI website (http://www.ncbi.nlm.nih.gov/geo/; accession number: GSE74106; Additional file 1).

**Fig. 1:**
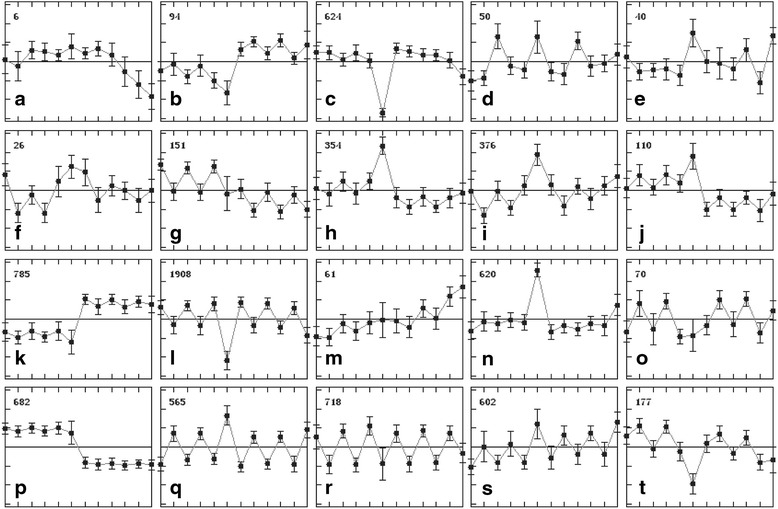
Expression pattern clustering of the differential genes in 12 samples. The points in each panel are from left-to-right: T6C, T24C, T6N, T24N, T6P, T24P, R6C, R24C, R6N, R24N, R6P and R24. **a**-**t** present 20 types of expression patterns of the differential genes that are classified by the software Multi Experiment Viewer

There were 8019 differentially-expressed probe sets [fold change (FC) ≥ 2] detected in this study. These included 3682 differential probe sets between different treatments, 3339 between different varieties (RH and TN1), and 5757 between different sampling times, with the two non-differentiating variables being the same (Additional file 2). To compare the gene expressional differences between RH and TN1, all 8019 differential probe sets were clustered into different groups based on their expression patterns using Multi-Experiment Viewer (Fig. 1). Significantly, 785 differential probe sets in Group k had low expression levels in TN1 and high expression levels in RH under all of the conditions; in contrast, 682 differential probe sets in Group p had low expression levels in RH and high expression levels in TN1 under all of the conditions. Therefore, Groups k and p roughly represent the genetic background differences between RH and TN1. Genes related to the constitutive resistance of RH should be included in these two groups. Groups a and m represent probe sets with BPH infestation-specific down- (6 differential probe sets) and up-regulated (61 differential probe sets) expression levels in RH, respectively. Genes in these two probe sets may be related to the BPH infestation-responsive resistance of RH. Differential probe sets in Groups l, q, r and s showed regular fluctuations associated with sampling time, indicating that their expression is mainly controlled by sampling time (circadian rhythm or other environmental factors). These four types of expression patterns included a total of 3793 differential probe sets, accounting for almost a half of the 8019 differential probe sets.

### Best reference genes for BPH resistance research

**Fig. 2:**
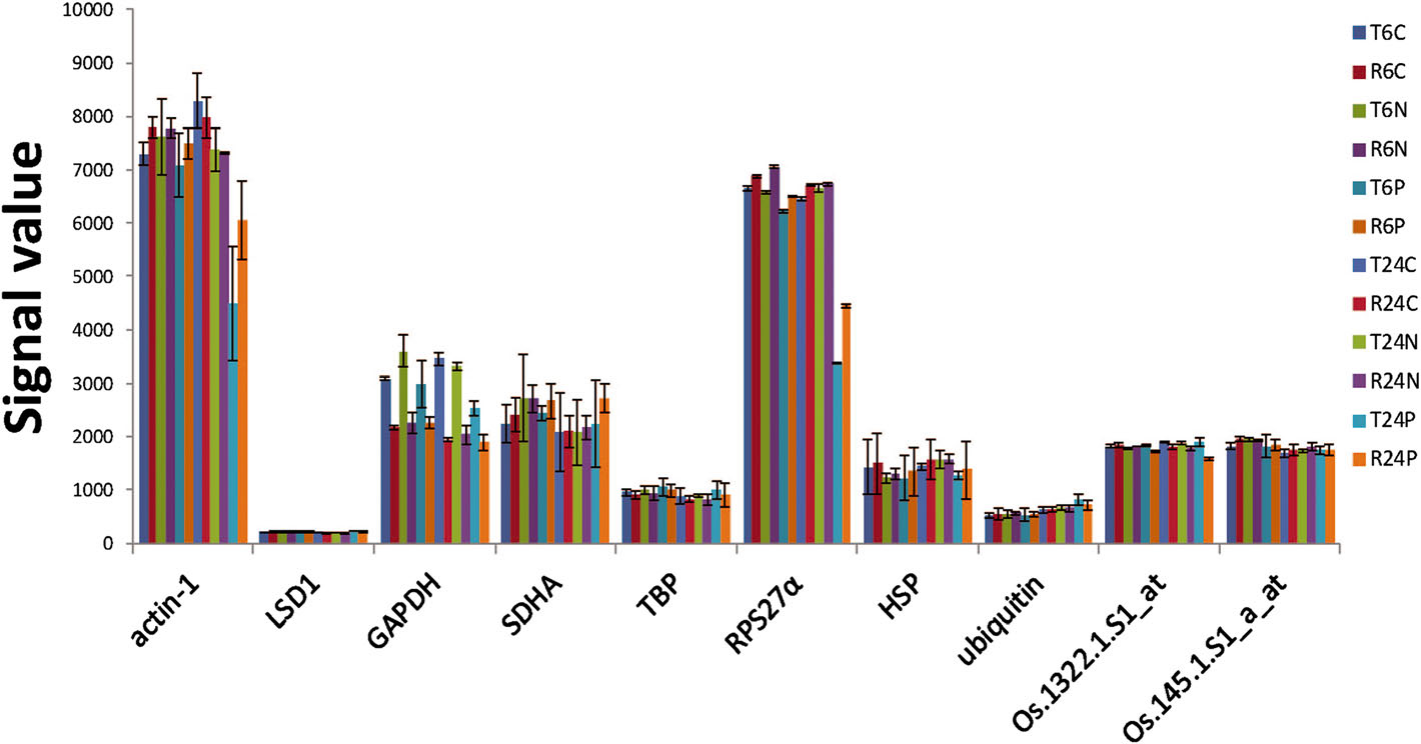
Signal values of *actin-1*, *LSD1*, *GAPDH*, *SDHA*, *TPB*, *RPS27α*, *HSP*, *Ubiquitin*, Os.1322.1.S1_at and Os.145.1.S1_a_at in the microarray data. Among the eight frequently-used reference genes, *TBP* and *Ubiquitin* were more stably expressed than the others under BPH infestation. Os.1322.1.S1_at (*Os05g23860*) and Os.145.1.S1_a_at (*Os02g56000*) are two newly identified references with stable expressions under BPH infestation. *Error bars* indicate standard deviations of three biological replicates

Reference genes are key factors that ensure the accuracy of a gene expressional analysis under specific conditions. It was very important to select a stably expressed gene under BPH infestation as the reference for quantitative reverse transcription polymerase chain reaction (qRT-PCR) analysis [22–24]. Figure 2 shows eight frequently-used reference genes, *actin-1* (*Os03g50890*), *LSD1* (*Os12g41700*), *GAPDH* (*Os02g38920*), *SDHA* (*Os07g04240*), *TPB* (*Os02g45410*), *RPS27α* (*Os01g22490*), *HSP* (*Os03g31300*) and *Ubiquitin* (*Os03g03920*), and their respective signal values extracted from our microarray data under different treatments. *Actin-1* and *RPS27α* were down-regulated in both TN1 and RH within 24 h after BPH infestation; *GAPDH* showed an obvious expressional difference between RH and TN1; and the expression level of *LSD1* was stable but relatively low. Among the remaining four references, *TBP* and *Ubiquitin* showed better overall consistency compared with *HSP* and *SDHA*. Therefore, *TBP* and *Ubiquitin* were determined to be suitable reference genes for BPH resistance research. *Ubiquitin* was used as the reference gene for all of the qRT-PCR analysis in this study.

To find additional stable reference genes for the gene expression analysis under BPH-infestation conditions, 373 probe sets with total variance < 5% in the microarray data were screened. Two probe sets with low expressional variances and appropriate expressional levels were selected, Os.1322.1.S1_at (*Os05g23860*) and Os.145.1.S1_a_at (*Os02g56000*) (Fig. 2), and the genes corresponding to these two probe sets were annotated to be rab GDP dissociation inhibitor alpha and 26S protease regulatory subunit 6A, respectively, in the MSU Rice Genome Annotation Project Release 7 (http://rice.plantbiology.msu.edu/index.shtml). They were also stably expressed during the entire life cycles of the *indica* rice varieties ‘Minghui 63’ and ‘Zhenshan 97’ [23].

### Different defense responses, at the genome level, between RH and TN1 to BPH infestation

At 6 h after needle puncturing, there were 92 differential probe sets in RH compared with the samples under natural growth, while there were 211 in TN1, indicating TN1’s more sensitive reaction to simple mechanical damage than RH. However, at 24 h after needle puncturing, fewer differential probe sets were detected in both RH (27) and TN1 (38) (Table 1), indicating that the reactions to simple mechanical damage had almost recovered by 24 h.

**Table 1:** Number of differentially-expressed probe sets detected in RH and TN1 under needle-puncturing and BPH-infestation treatments

Comparison	FC^a^ > 2		FC^a^ > 5		Total
	Up	Down	Up	Down	
R6N/R6C	69	10	13	0	92
T6N/T6C	164	21	26	0	211
R6P/R6C	126	48	33	2	209
T6P/T6C	151	10	12	0	173
R24N/R24C	26	0	1	0	27
T24N/T24C	31	3	4	0	38
R24P/R24C	405	136	72	1	614
T24P/T24C	1368	1639	301	48	3356

^a^FC indicates the fold change of differential probe sets under different treatments

At 6 h after BPH infestation, there were more differential probe sets in RH (209) than in TN1 (173). The number of differential probe sets with high FCs (>5) was 35 in RH and 12 in TN1 (Table 1). Although TN1 was relatively more sensitive to simple mechanical damage, more differential probe sets were detected in RH at 6 h (early stage) after BPH infestation, implying that the defense responses to BPH infestation in RH was more intensive than in TN1. At 24 h after BPH infestation, more differential probe sets (614) were detected in RH compared with at 6 h, and the number of probe sets with FCs > 5 also increased from 35 to 73, indicating that the signals of BPH infestation were progressively transmitted to the related downstream genes. However, 24 h after BPH infestation, the number of differential probe sets in TN1 dramatically increased to 3356, and the number with FCs > 5 also increased dramatically from 12 to 349 (Table 1). Thus, more differential probe sets were detected in RH at 6 h after BPH infestation; however, many more differential probe sets were detected in TN1 at 24 h after BPH infestation. Many of the differential probe sets detected in TN1 at 24 h after BPH infestation may have represented the responses to wounding and various physiological stresses resulting from the serious damage caused by the BPH, instead of a resistance response. In RH, the majority of differential probe sets were up-regulated at either 6 or 24 h after BPH infestation, indicating that the inducible expression of resistance-related genes may be the main molecular basis of the defense responses in RH to BPH.

**Fig. 3:**
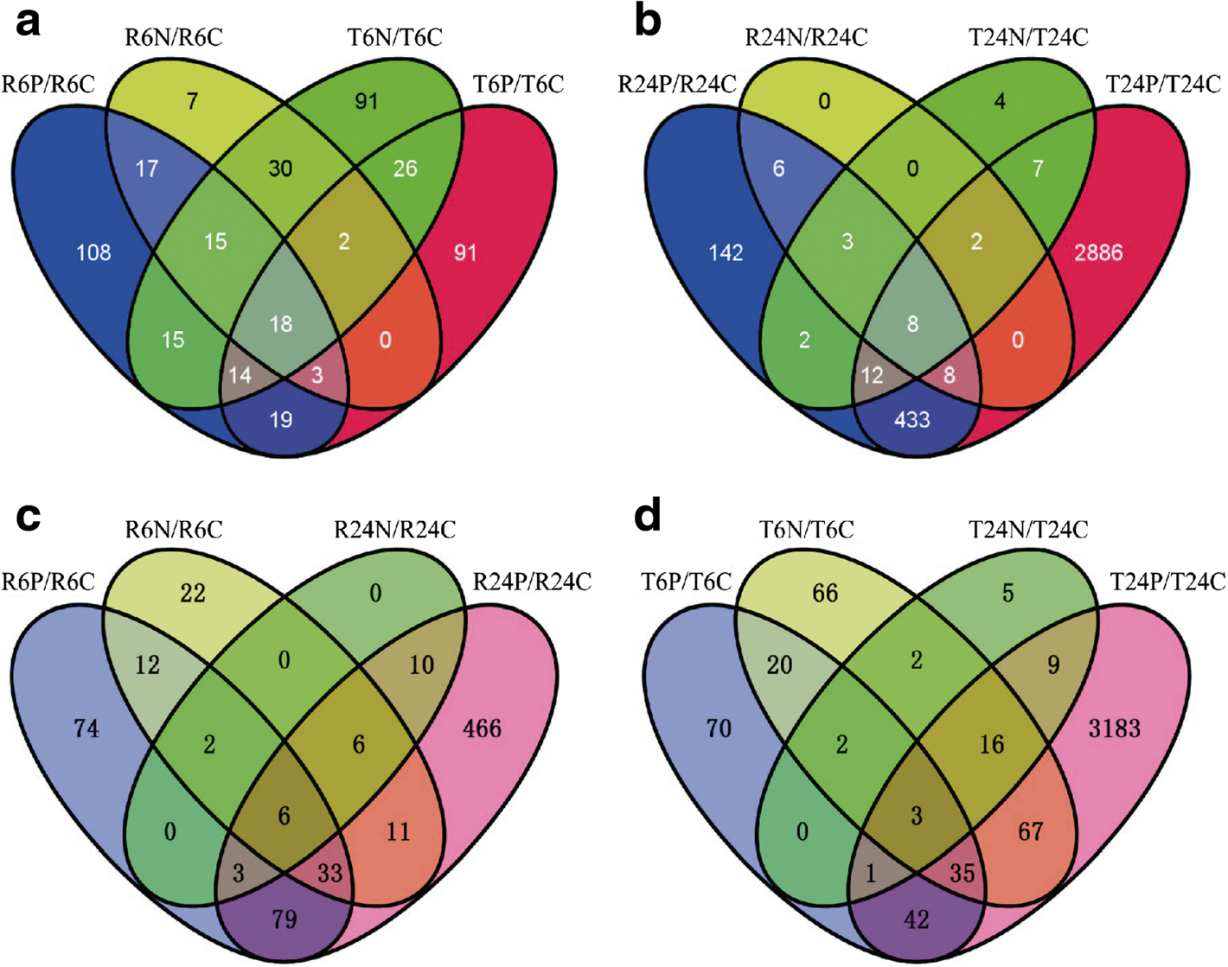
Venn diagram analysis of differential probe sets under needle-puncturing and BPH infestation treatments at different times. **a** 6 h after needle puncturing or BPH infestation in RH and TN1; **b** 24 h after needle puncturing or BPH infestation in RH and TN1; **c** 6 and 24 h after needle puncturing or BPH infestation in RH; and **d** 6 and 24 h after needle puncturing or BPH infestation in TN1

The Venn diagram analysis of differential probe sets is shown as Fig. 3. Among the differential probe sets of RH at 6 h after BPH infestation, 53 were also present at 6 h after needle puncturing. Among the differential probe sets of TN1 at 6 h after BPH infestation, 60 were also present at 6 h after needle puncturing. At 24 h after the treatments, most of the differential probe sets under needle puncturing, in either RH or TN1, were also present under BPH infestation. By excluding the differential probe sets of RH and TN1 under needle puncturing and those of TN1 under BPH infestation, a total of 192 RH-specific and BPH-inducible probe sets were determined. These 192 probe sets may represent the basis of the inducible defense in RH to BPH (Additional file 3).

### Gene Ontology (GO) analysis

**Fig. 4:**
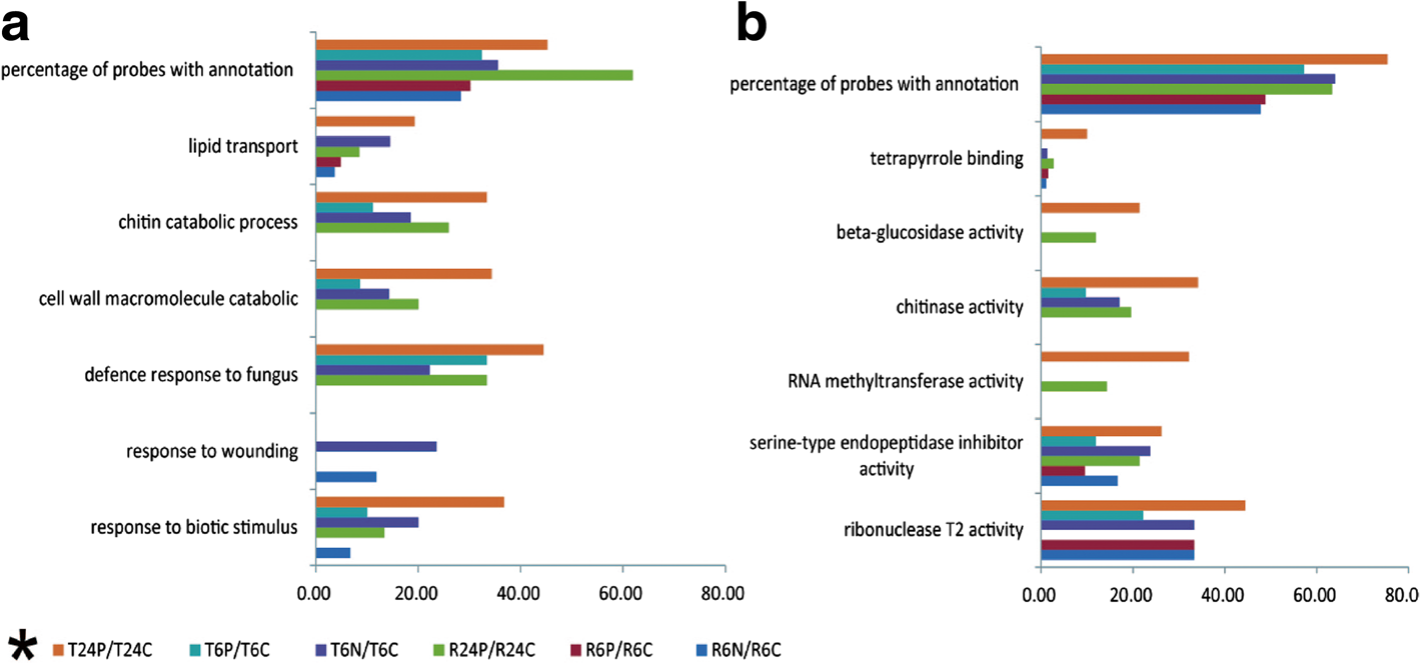
Percentage of differential probe sets with annotations in the T24P/T24C, T6P/T6C, T6N/T6C, R24P/R24C, R6P/R6C and R6N/R6C groups. **a** biological process; and **b** molecular function

The differential probe sets of RH and TN1 under needle puncturing (the differential probe set groups of R6N/R6C and T6N/T6C) or BPH infestation (groups of R6P/R6C, R24P/R24C, T6P/T6C and T24P/T24C) were subjected to GO analysis using GOEAST software, which includes two principal GO categories: biological process (BP) and molecular function (MF) (Additional file 4). The GO analysis of the groups R24N/R24C and T24N/T24C was not conducted because very few differential probe sets were identified at 24 h after needle puncturing. The first level of BP categories and MF categories with significant differences and the percentages of related differential probe sets with annotations are shown in Fig. 4. As expected, in the needle-puncturing groups (R6N/R6C and T6N/T6C), ‘response to wounding’ was the GO term found having significant differences. Of the BP categories, the differential probe sets of ‘response to biotic stimulus’, ‘response to abiotic stimulus’, ‘response to wounding’, ‘defense response to fungus’, ‘cell wall macromolecule catabolic’ and ‘chitin catabolic process’ were mainly up-regulated, while those of ‘lipid transport’ were mainly down-regulated (Fig. 4a). Of the MF categories, the differential probe sets of resistance-related projects, such as ‘serine-type endopeptidase inhibitor activity’, ‘chitinase activity’, ‘beta-glucosidase activity’ and ‘tetrapyrrole binding’ were mainly up-regulated, while those of ‘RNA methyltransferase activity’ were mainly down-regulated (Fig. 4b).

### Seeking BPH resistance-related gene candidates in RH

Because too many differential probe sets were identified to test them individually in this study (Fig. 1), strategies were employed to reduce the number of BPH resistance-related gene candidates. One strategy was to combine the microarray analysis with QTL mapping. Four BPH-resistance QTLs were previously reported [25–27] (Additional file 5). In this study, we identified 106 differential genes between RH and TN1 that were located within the four BPH-resistance QTL regions (Additional file 6), and 14 of them are likely to be associated with the BPH resistance according to their functional annotations (Table 2). Among them, five genes were located in the *Qbph3* region, five in *Bph17*, one in *Bph3* and three in the *Qbph10* region. A major BPH-resistance QTL, *BPH17/BPH3*, which was mapped in the *Bph17* region, was cloned and contains three tandem genes encoding lectin receptor kinases (*OsLecRK1–OsLecRK3*) [9]. However, the expression levels of these three genes were too low to be detected in this study.

**Table 2:** Fourteen selected candidate genes in the four BPH-resistance QTLs of RH

Candidate gene	QTL	Gene annotation
Os03g12260	*Qbph3*	Cytochrome P450 family protein, expressed
Os03g12500	*Qbph3*	Cytochrome P450 74A2, putative, expressed
Os03g12660	*Qbph3*	Cytochrome P450 family protein, expressed
Os03g13370	*Qbph3*	TPR Domain containing protein, expressed
Os03g16740	*Qbph3*	Protein kinase APK1B, chloroplast precursor, expressed
Os04g08390	*Bph17*	Leucine Rich Repeat family protein, expressed
Os04g09920	*Bph17*	Cytochrome P450 family protein, expressed
Os04g11780	*Bph17*	resistance protein LR10, putative
Os04g14220	*Bph17*	NB-ARC domain containing protein, expressed
Os04g15650	*Bph17*	Leucine Rich Repeat family protein, expressed
Os06g04080	*Bph3*	Glycosyl hydrolases family 17 protein, expressed
Os10g39930	*Qbph10*	Cytochrome P450 family protein, expressed
Os10g41290	*Qbph10*	Protein kinase PVPK-1, putative, expressed
Os10g41550	*Qbph10*	Glycosyl hydrolase family 14 protein, expressed

**Fig. 5:**
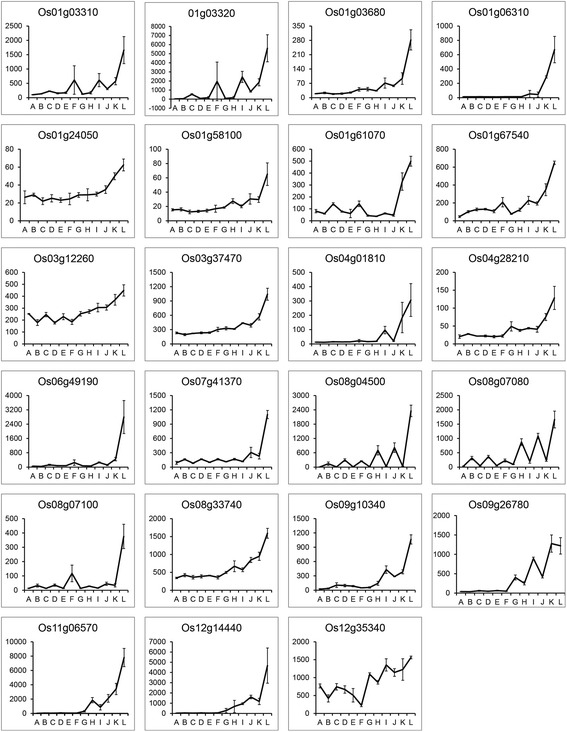
Expression patterns of 23 selected BPH resistance-related gene candidates. These gene candidates share a similar expression pattern, which was inducible by BPH infestation only in RH but not by mechanical damage or in TN1. The six genes in boldface were selected for further detection. A–L on the *x*-axis represent T6C, R6C, T24C, R24C, T6N, R6N, T24N, R24N, T6P, R6P, T24P and R24P, respectively. *Error bars* indicate standard deviations of three biological replicates

The second strategy was to determine BPH resistance-related gene candidates by examining gene expression patterns. Clustering the gene expression patterns divided the differential probe sets into 20 groups (Fig. 2). Group m represented those differential probe sets that were BPH-inducible only in RH but not in TN1. A Venn diagram analysis also helped to remove those differential probes sets induced by both mechanical damage and BPH infestation (Fig. 3). Finally, 23 gene candidates were selected based on the expression pattern clustering and Venn diagram analysis. These selected gene candidates shared a similar expression pattern (Fig. 5). They were inducible by BPH infestation only in RH, but not by mechanical damage or in TN1. Among the 23 BPH resistance-related gene candidates, *Os03g12660*, which encodes a cytochrome P450 protein, was the only gene also located in a BPH resistance QTL region (*Qbph3*).

### Dynamic changes of SA and JA levels and differential genes associated with SA, JA and ET pathways under BPH infestation

**Fig. 6:**
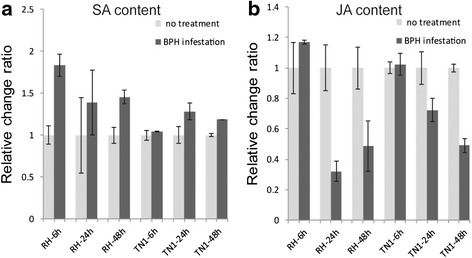
SA and JA relative change ratios in RH and TN1 under BPH infestation. **a** SA relative change ratios in RH and TN1; **b** JA relative change ratios in RH and TN1. *Error bars* indicate standard deviations of three biological replicates

The SA and JA levels (free status) in RH and TN1 were determined at 6, 24 and 48 h after BPH infestation. Overall, the SA level in both RH and TN1 increased significantly after BPH infestation (Fig. 6a); while the JA level in both RH and TN1 decreased significantly after BPH infestation (Fig. 6b). Moreover, the change in the SA content in RH occurred more rapidly than that in TN1. The SA content in RH had increased at 6 h after BPH infestation, while that in TN1 did not increase until 24 h (Fig. 6a).

All of the differential genes under BPH-infestation treatment involved in the SA, JA and ET pathways are listed in Table 3. The information concerning the genes involved in the SA, JA and ET pathways refer to Kyoto Encyclopedia of Genes and Genomes annotations. Six differential genes involved in SA metabolism were detected, and they were up-regulated, except for one gene (*Os02g19970*) that was involved in both ET and SA metabolism.

**Table 3:** Differentially-expressed genes involved in the SA, JA and ET pathways during BPH infestation

Gene ID	Fold changed (under BPH infestation)				Gene annotation
	R6P/R6C	R24P/R24C	T6P/T6C	T24P/T24C	
SA pathway(59)					
Os02g19970	0.41	-	-	-	aminotransferase, classes I and II, domain containing protein, *OsNAAT2*
Os03g13210	-	-	9.43	-	peroxidase precursor
Os04g43800	3.08	2.44	-	-	phenylalanine ammonia-lyase, *OsPAL6*
Os08g02110	3.57	3.29	5.12	-	peroxidase precursor
Os08g34790	-	-	4.08	-	AMP-binding domain containing protein, *Os4CL5*
Os11g02130	2.07	-	2.60	-	peroxidase precursor
JA pathway(42)					
Os01g06600	-	-	2.50	-	glutaryl-CoA dehydrogenase, mitochondrial precursor
Os01g55650	-	-	2.00	-	phospholipase, patatin family, *OspPLAIVα*
Os02g10120	-	-	3.25	-	lipoxygenase, *OsLOX1*
Os02g17390	-	-	2.07	-	3-hydroxyacyl-CoA dehydrogenase
Os05g07090	-	-	4.93	-	acyl-coenzyme A dehydrogenase, mitochondrial precursor
Os06g11210	-	-	4.09	-	12-oxophytodienoate reductase
Os08g39840	-	-	6.92	-	lipoxygenase, chloroplast precursor, *OsLOX9*
Os08g39850	-	-	6.19	-	lipoxygenase, chloroplast precursor, *OsLOX8*
Os11g39220	-	-	2.37	-	acyl-coenzyme A oxidase, *OsACX2*
Os12g26290	-	-	2.93	-	alpha-DOX2
Os12g37260	-	-	3.64	-	lipoxygenase 2.1, chloroplast precursor, *OsLOX11*
Os03g08310	3.32	5.89	-	-	ZIM domain containing protein, OsJAZ9; OsTIFY11a
Os03g08320	8.16	8.55	13.67	4.82	ZIM domain containing protein, OsJAZ11; OsTIFY11c
Os03g08330	2.65	3.03	-	-	ZIM domain containing protein, OsJAZ10; OsTIFY11b
Os09g26780	4.82	3.15	-	-	zinc-finger protein, OsJAZ8; OsTIFY10c
Os10g25230	3.40	4.52	-	-	ZIM domain containing protein, OsJAZ13; OsTIFY11e
Os10g25290	3.21	2.73	2.93	3.12	ZIM domain containing protein, OsJAZ12; OsTIFY11d
ET pathway(82)					
Os01g52260	-	-	2.85	2.10	serine acetyltransferase protein
Os01g59920	-	-	0.33	-	cysteine synthase, chloroplast precursor
Os01g74650	-	-	0.46	-	cysteine synthase, mitochondrial precursor
Os02g14110	-	-	0.47	-	aminotransferase, classes I and II, domain containing protein
Os02g19970	-	-	0.41	-	aminotransferase, classes I and II, domain containing protein, *OsNAAT2*
Os02g53180	-	-	3.30	-	1-aminocyclopropane-1-carboxylate oxidase protein, *OsACO3*
Os03g25940	0.38	-	-	-	cystathionine gamma-synthase
Os03g55280	-	-	0.25	-	semialdehyde dehydrogenase, NAD binding domain containing protein
Os06g36880	-	-	2.22	-	cysteine synthase
Os07g08500	-	-	0.41	-	C-5 cytosine-specific DNA methylase, *OsMET1b*
Os07g22600	-	-	0.49	-	spermidine synthase
Os08g25390	0.47	-	0.22	-	bifunctional aspartokinase/homoserine dehydrogenase, chloroplast precursor
Os09g12290	-	-	0.41	-	bifunctional aspartokinase/homoserine dehydrogenase, chloroplast precursor
Os09g27750	-	-	2.27	-	1-aminocyclopropane-1-carboxylate oxidase 1, *OsACO2*; *OsACO1*
Os10g01570	-	-	0.24	-	C-5 cytosine-specific DNA methylase, OsCMT3a
Os10g37340	2.19	2.10	2.27	-	cystathionine gamma-synthase

Among 42 JA pathway-related genes, 17 were up-regulated under BPH infestation in either RH or TN1. However, the expression level patterns of differential genes involved in the JA pathway were diverse between RH and TN1 (Table 3). All six differential genes in RH were up-regulated and were jasmonate ZIM-domain (JAZ) transcription factors, which are important negative regulators in the JA signal transduction pathway, implying that the JA pathway was repressed in RH under BPH infestation (Table 3). All 11 differentially expressed genes only presented in TN1 under BPH infestation were JA metabolism-related genes, including several JA synthesis-related lipoxygenase (LOX) genes. Additionally, 16 differential genes related to ET metabolism were detected. Most of them were present in TN1 and were down-regulated at 24 h after BPH infestation (Table 3). Comparatively, the ET pathway in RH was less affected after BPH infestation.

### Isolating BPH-inducible promoters

**Fig. 7:**
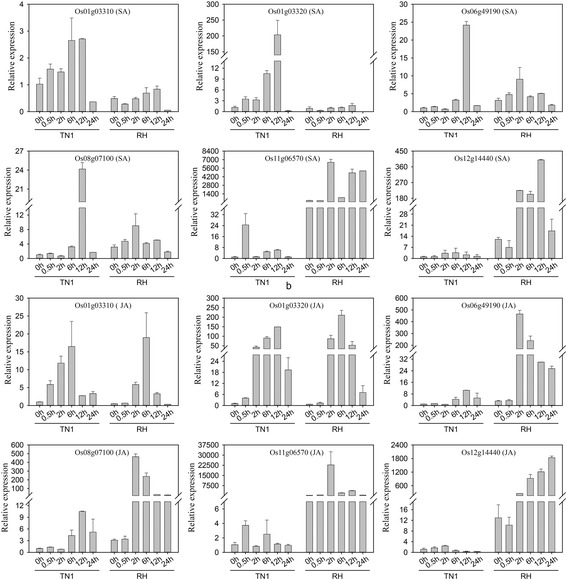
Expression pattern testing of six selected BPH resistance-related genes in RH and TN1 after independent SA and JA treatments. All six genes were up-regulated after independent SA and JA treatments. The gene expression levels in TN1 without a treatment were normalized as the calibrators. *Error bars* indicate standard deviations of three biological replicates

Microarray analysis facilitates the isolation of BPH-inducible promoters. Six representative BPH-inducible genes were selected from 23 BPH resistance-related gene candidates (Fig. 5). The expression levels of these six genes were only induced by BPH infestation in RH, and they were only slightly, or not at all, induced by needle puncturing or in TN1. A qRT-PCR analysis showed that these six genes were also up-regulated by a SA or JA treatment (Fig. 7).

The genomic sequences, including the promoter regions and coding sequences of these six selected BPH-inducible genes, were isolated by PCR from both RH and TN1, and then subjected to sequencing. No significant sequence differences in the promoter regions of these six genes were found between RH and TN1. Furthermore, a DNA sequence comparison of the coding regions showed no differences between RH and TN1. It was hypothesized that the inducibility of these genes in RH was not determined by the promoter sequences, but by the genetic background.

Additional rice varieties, including four BPH-resistant (‘PTB33’, ‘R644’, ‘B5’ and ‘IR54751’) and four BPH-susceptible varieties (‘Bai56’, ‘Nipponbare’, ‘Zhenshan97B’ and ‘9311’), were used for further examination. Similar to in RH and TN1, the expression levels of the six selected genes were relatively high and significantly induced by BPH infestation in all of the resistant varieties, but was relatively low and barely induced by BPH infestation in all of the susceptible varieties (Additional file 7). The genomic sequences of these six resistance-related genes were also isolated from all eight additional rice varieties and then compared. Neither the promoters nor the coding sequences had any significant sequence differences between the resistant and susceptible varieties.

## Discussion

In this study, the whole genome-wide responses of the resistant RH and the susceptible TN1 under BPH infestation were determined and compared through a microarray analysis. A similar study reported by Wang et al., conducted a microarray analysis using RH and TN1 as the rice materials under infestation [28, 29]. However, the study of Wang et al. did not incorporate an untreated control for each sampling time point (0, 8 and 24 h after BPH infestation), and they simply used the samples collected at 0 h as the control for the microarray analysis. This design is problematic because the expression levels of many genes are regulated by a circadian rhythm or other environmental factors. The expression pattern clustering in our study clearly showed that 3793 differential probe sets, such as groups l, q, r and s in Fig. 2, accounting for almost a half of all detected differential probe sets, were obviously affected by a circadian rhythm or other environmental factors. These were possibly mistaken for having an association with BPH infestation because of the lack of a control for each sampling time. Second, our study included the needle-puncturing treatment, while the study of Wang et al. did not. The immune responses of rice to BPH involve molecular interactions between rice and BPH, and the resistance reactions of rice are triggered by specific effectors in BPH saliva, which are delivered into rice cells by feeding and are recognized by specific receptors in rice [30]. The needle-puncturing treatment can be used to exclude those differential genes that respond to simple mechanical wounding.

There are several other studies that also profiled gene expression in rice varieties responding to BPH infestation using RNA-sequencing (RNA seq) or microarray analyses [31, 32]. In the study of Lv et al., RNA-seq of BPH susceptible WT ‘9311’ and resistant ‘9311’ introgression lines containing *BPH15* was conducted to help identify candidate genes of *BPH15*, which are located in a recombination cold spot [31]. The study of Wang et al. compared gene expression profiles between *Bt* and non-*Bt* rice in response to BPH infestation [32]. In these two studies, the genetic backgrounds of rice materials used for the gene expressional analyses were both BPH susceptible ‘9311’ [31] or ‘Xiushui 11’ [32]; therefore, their results could not present the responses of a rice variety resistant to BPH infestation .

The microarray analysis showed significant response differences between RH and TN1 to BPH infestation. The response of RH was more active at the early stage of BPH infestation compared with TN1 because more differential probe sets (209) were detected in RH at 6 h after BPH infestation than in TN1 (173). In contrast, many more differential probe sets (3356) were detected in TN1 at 24 h after BPH infestation than in RH (614). Many of the differential probe sets presented at 24 h reflected the physiological and metabolic changes in TN1 under BPH attack caused by its lack of resistance. The GO analysis also confirmed that many of the differential probe sets in TN1 at 24 h after BPH infestation were related to abiotic and biotic stresses. Most differential probe sets detected in RH at either 6 or 24 h after BPH infestation were up-regulated, implying that the up-regulation of the defense-related genes was possibly the molecular basis of BPH resistance in RH.

The change in the SA level in RH occurred more rapidly than in TN1, and the increase in the SA level occurred prior to the decrease in the JA level, indicating that the SA pathway plays a leading role in triggering the BPH-resistance response. The decrease in the JA content was probably caused by the antagonist effect of SA, which increased after BPH infestation. The up-regulation of SA synthesis-related genes was the main change trend in the SA pathway, which was consistent with the change in the SA content after BPH infestation. However, differential genes in the JA pathway showed significant diverse change patterns between RH and TN1. In RH, all six differential genes were up-regulated and were JAZ transcription factors, which are important negative regulators of the JA pathway. While in TN1, 11 differential genes involved in JA synthesis were up-regulated and only two were JAZ transcription factors. JA is also associated with wounding responses. As a susceptible variety, TN1 lacks BPH resistance-related genes and undergoes much more serious damage under BPH infestation. Therefore, the up-regulation of JA synthesis-related genes probably represented a response to wounding or another stress caused by BPH, instead of a resistance response.

Although the four major BPH resistance QTLs were cloned by map-based techniques, many BPH resistance-related genes, including *OsHI-LOX* [12], *Bphi008a* [13], *OsERF3* [14], *OsPLD α4* and *α5* [15], *OsACS2* [17], *Osr9-LOX1* [19], and *OsJMT1* [20], were also isolated using a reverse genetics strategy. Almost all of these BPH resistance-related genes are inducible to BPH or SSB infestation, and are involved in SA, JA, or ET pathways. A cDNA microarray analysis of RH and TN1 under BPH infestation laid the foundations for identifying new BPH resistance-related genes.

BPH-responsive promoters have potential applications in developing BPH-resistant genetically modified rice varieties. Therefore, the approximate 2-kb upstream promoter regions of some representative RH-specific and BPH-inducible genes were isolated and fused with the GUS reporter gene. However, when tested in BPH-susceptible rice varieties, these putative BPH-inducible promoters were either not, or were poorly, induced under BPH infestation. A DNA sequencing analysis showed that these RH-specific and BPH-inducible genes showed no significant sequence differences in both the promoter and coding regions between BPH-resistant and -susceptible rice varieties. Thus, the promoters of most of these BPH-inducible genes were probably not BPH-inducible, and they may be regulated by their upstream trans-regulators in the BPH-resistance network. In other words, their expression pattern was determined by the genetic background instead of the promoter sequence.

## Conclusions

In this study, a cDNA microarray analysis was conducted to reveal the genome-wide response differences between RH and TN1 under BPH infestation. Expression pattern clustering of differential probe sets demonstrated that 1467 differential probe sets (Groups p and k) may be associated with the constitutive resistance of RH and 67 (Groups a and m) with the BPH infestation-responsive resistance. The Venn diagram analysis determined 192 RH-specific and BPH-inducible probe sets. Finally, 23 genes were selected as BPH resistance-related gene candidates based on the expression pattern clustering and Venn diagram analysis. The overall response of RH to BPH infestation was more prompt than that of TN1, at the global gene expression and the SA levels. In RH, the significant increase in the SA (6 h after BPH infestation) occurred prior to the significant decrease in the JA (24 h after BPH infestation), implying a leading role of SA in mediating BPH-resistance responses. In RH, differential genes in the SA pathway were synthesis-related and up-regulated after BPH infestation. The differential genes in the JA pathway were up-regulated and were JAZ transcription factors, which are important negative regulators of the JA pathway. These results were consistent with the changes in the SA and JA levels in RH seedlings infested with BPH. Comparatively, genes involved in the ET pathway were less affected by a BPH infestation in RH. The results of this study aid in understanding the molecular foundation of BPH resistance in the RH and facilitate the identification of new resistance-related genes for breeding BPH-resistant rice varieties.

## Methods

### Plant materials

Rice varieties RH and TN1 were kindly provided by Prof. Hongxia Hua. RH is an indica rice cultivar from Sri Lanka, with a high-level, broad-spectrum resistance to all BPH biotypes. The indica rice cultivar TN1 is highly susceptible to all BPH biotypes. Four BPH-resistant rice varieties ‘PTB33’, ‘R644’, ‘B5’ and ‘IR54751’ were identified and provided by Prof. Yuqing He. Four BPH-susceptible rice varieties ‘Bai56’, ‘Nipponbare’, ‘Zhenshan97B’ and ‘9311’ were previously collected and maintained in our laboratory. The BPH resistance of all involved rice materials were validated in our laboratory prior to the current research.

### Plant sample preparation for the microarray analysis

Two rice varieties, BPH-resistant RH and BPH-susceptible TN1, were used. The BPH population for this experiment was collected from a local rice field in Wuhan, China, and raised on TN1 in cages. This BPH population is a mix of BPH biotypes 1 and 2, which represent the main natural BPH populations present locally.

Seeds of RH and TN1 were sown in small pots and grown under natural conditions. Approximately 15 seeds were sown per pot, and after 2 weeks only 10 well-grown rice seedlings were retained in each pot. Three treatments, untreated (naturally grown), needle puncturing and BPH infestation, were implemented on the 2-week-old RH and TN1 seedlings. For needle puncturing, each plant was pricked with a needle 15 times at the bottom of the seedling, and then grown normally. For BPH infestation, approximately 100 third-instar BPH nymphs were introduced per pot (an average of 10 nymphs per seedling). Each pot was placed in a single netted cage to prevent nymphs from escaping. Sampling time points of 6 and 24 h after BPH infestation were used for all of the treatments. There were three replications for each treatment and sampling time point. All 10 seedlings from each pot were collected as a replication. The seedling shoots (the aerial part) were collected after the treatment, immediately placed in liquid nitrogen and then stored at –80 °C. Finally, all of the samples were shipped to CapitalBio Corporation (Beijing, China) for the microarray determinations.

### RNA extraction, microarray hybridization and data analysis

RNA extraction, purification, microarray hybridization and gene annotation were conducted by CapitalBio. After RNA extraction, the total RNA was purified using a Nucleospin RNA Clean-up kit (Macherey-Nagel, Germany) following the manufacturer’s instructions. The RNA quality was determined by electrophoresis on 1% agarose gel with formaldehyde. Biotin-labeled cRNA was synthesized using a MessageAmp™ II-Biotin kit (Ambion, TX, USA). Under the guidance of the Affymetrix GeneChip Expression Analysis technical manual, the biotin-labeled cRNA was linearized into fragments of 35–300 bp in length. The linear cRNA was hybridized with an Affymetrix Rice Genome array in an Affymetrix GeneChip Hybridization Oven 320 at 45 °C for 16 h. After hybridization, the chip was washed and dyed in an Affymetrix Fluidics Station 400. Finally, the chip was scanned using a GeneChip Scanner 3000. The Single Array Analysis, Comparison Analysis and Molecule Annotation System were performed by CapitalBio.

### Data analysis

Venn diagrams were made on the website http://bioinfogp.cnb.csic.es/tools/venny/. Probe-set expression pattern clustering was performed using the Multi Experiment Viewer software in the java environment (http://mev.tm4.org/). The GO analysis was performed on the website http://omicslab.genetics.ac.cn/GOEAST/php/affymetrix.php?#step1_anchor [33]. All of the expression-differential probes were previously identified by CapitalBio using a one-way ANOVA. The gene information used in our analysis was from the websites Kyoto Encyclopedia of Genes and Genomes (http://www.kegg.jp/kegg/kegg2.html), CREP (http://crep.ncpgr.cn/), GRASSIUS (http://grassius.org/grasstfdb.html) and RGAP (http://rice.plantbiology.msu.edu/index.shtml).

### Expression profiling following plant hormone treatments

The RH and TN1 plants were grown under greenhouse conditions at 25–30 °C and a photoperiod of 14 h light/10 h dark. At the trefoil stage, the RH and TN1 plants were transferred into hydroponic nutrient solutions containing either 0.1 mM SA or JA. At each time point of 0, 0.5, 2, 6, 12 and 24 h after treatment, three to five whole plants were collected for RNA isolation and extraction.

### qRT-PCR analysis

RT with 2 μg of DNase-treated total RNA was performed using a Transcriptor First Strand cDNA Synthesis Kit (Roche, Mannheim, Germany) following the manufacturer’s instructions. The real-time PCR was conducted using SYBR Premix Ex Taq TM (Takara, Dalian, China). The reactions were prepared in a volume of 20 μL containing 10 μL of SYBR Premix Ex Taq TM (2×), 0.4 μM for each gene-specific primer (10 μM), 0.4 μL of ROX Reference Dye II (10 μM), 2 μL of cDNA template and 6.8 μL of ddH_2_O. The *Ubiquitin* gene was used as the reference gene. The real-time PCR was executed on a 7500 Real-time PCR System (Applied Biosystems, Foster City, CA, USA). Each RNA sample was used twice in technical replications. All of the primers used in this study are listed in Additional file 8.

### Hormone determinations

SA and JA levels in plant tissues were measured by liquid chromatography-tandem mass spectrometry (LC-MS/MS 8030 plus, Shimadzu, Beijing, China) as follows: a total of 200 mg of fresh sample frozen in liquid nitrogen was well ground using a small glass pestle in a 2-mL vial. Following the addition of 1.0 mL of 80% methanol, homogenates were well mixed in an ultrasonic bath and then kept overnight at 4 °C. After being centrifuged at 15,200 × *g* for 10 min, the supernatant was collected and then vacuumed to dryness in a Jouan RCT-60 concentrator. Dried extract was dissolved in 200 μL of 0.1 mol/L sodium phosphate solution (pH 7.8) and later passed through a Sep-Pak C18 cartridge (Waters, MA, USA). The cartridge was eluted with 1.5 mL of 80% methanol, and the eluate was vacuumed to dryness again. After being dissolved in 10 mL of 10% methanol, 5 μL of such solution was injected into the LC-MS/MS system. LC was performed using a 2.0 mm I.D. × 75 mm Shim-pack XR ODSI column (2.2 μm, Shimadzu) at a column temperature of 40 °C. The mobile phase, containing solvent A (0.02% v/v aqueous acetic acid) and solvent B (100% v/v methanol), was employed in a gradient mode [time/A concentration/B concentration (min/%/%) for 0/90/10; 5/10/90; 6/10/90 and 6.1/90/10] at an eluent flow rate of 0.3 mL/min. For SA, the mass system was set to multiple reactions monitoring mode using electrospray ionization in the negative ion mode. The operation conditions used were a nebulizing gas flow of 2.5 L/min, drying gas flow of 15 L/min, desolvation temperature of 150 °C and heat block temperature of 400 °C. The ionization conditions were pre-bias voltages of 10 V for quadrupole 1 and 24 V for quadrupole 3, collision energy of 28 eV, and mass-to-charge ratio of 137/93. For JA, the mass system was set to multiple reactions monitoring mode using electrospray ionization in the negative ion mode. The operation conditions used were a nebulizing gas flow of 3 L/min, drying gas flow of 15 L/min, desolvation temperature of 250 °C and heat block temperature of 500 °C. The ionization conditions were pre-bias voltages of 10 V for quadrupole 1 and 10 V for quadrupole 3, collision energy of 15 eV, and mass-to-charge ratio of 209/59.

## Additional files

Additional file 1: Raw data of all detected probe sets (57,194) in 36 cDNA chips (https://www.ncbi.nlm.nih.gov/geo/, GEO accession number: GSE74106). (TXT 1023 kb)
Additional file 2: Information on the 8019 differentially-expressed probe sets detected in this study. (XLSX 258 kb)
Additional file 3: Information on 192 RH-specific and BPH-inducible differentially-expressed probe sets. (XLSX 12 kb)
Additional file 4: GO analysis of differential probe sets between RH and TN1 under needling puncturing and BPH infestation conditions. (XLSX 183 kb)
Additional file 5: Four rice BPH-resistance QTLs in chromosomes 3, 4, 6 and 10 of RH that were previously reported. (JPG 161 kb)
Additional file 6: The 103 potential resistance-related genes in the four BPH-resistance QTL regions in RH. (XLSX 15 kb)
Additional file 7: Expression pattern analysis of six selected BPH-induced genes in 10 rice varieties. The gene expression levels in TN1 with no treatment were normalized as the calibrators. Error bars indicate the standard deviations of three biological replicates. The expression levels of all the six selected genes were relatively high and significantly induced by BPH infestation (24 h) in the resistant varieties RH, ‘PTB33’, ‘R644’, ‘B5’ and ‘IR54751’, but were relatively low and barely induced by BPH infestation in the susceptible varieties TN1, ‘Bai56’, ‘Nipponbare’, ‘Zhenshan 97B’ and 9311. (JPG 2037 kb)
Additional file 8: All of the primers used in this study. (DOCX 22 kb)

## Abbreviations

BPH: Brown planthopper
ET: Ethylene
GO: Gene Ontology
JA: Jasmonic acid
JAZ: Jasmonate ZIM-domain protein
QTL: Quantitative trait locus
RH: Rathu Heenati
SA: Salicylic acid
TN1: Taichung Native 1
