# Supplementary figures and images for: Gene expression and plant hormone levels in two contrasting rice genotypes responding to brown planthopper infestation
Source: BMC Plant Biol. 2017 Feb 28;17:57. doi: 10.1186/s12870-017-1005-7 (PMC5331639; doi:10.1186/s12870-017-1005-7)

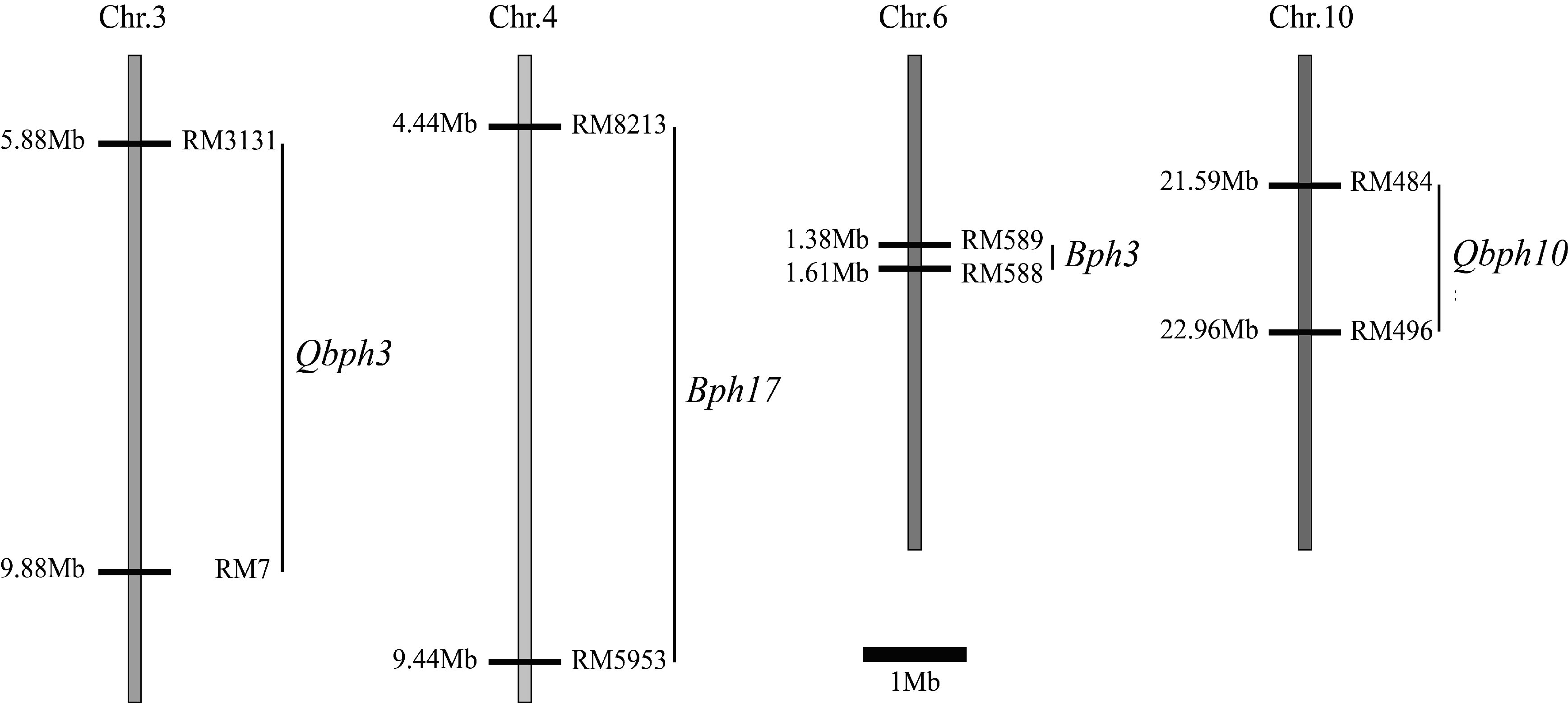

Supplement: Additional file 5: — Four rice BPH-resistance QTLs in chromosomes 3, 4, 6 and 10 of RH that were previously reported. (JPG 161 kb) [file 12870_2017_1005_MOESM5_ESM.jpg]

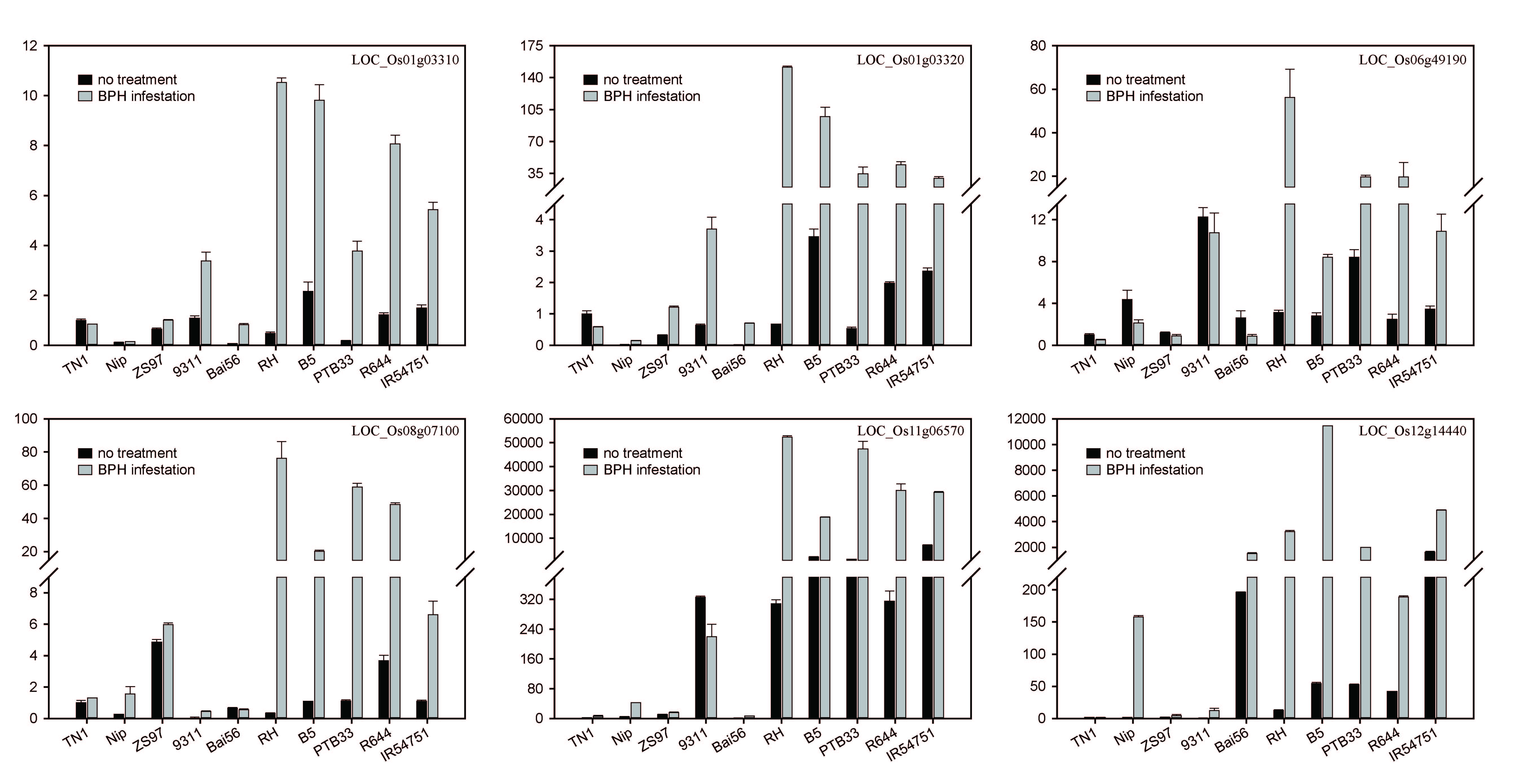

Supplement: Additional file 7: — Expression pattern analysis of six selected BPH-induced genes in 10 rice varieties. The gene expression levels in TN1 with no treatment were normalized as the calibrators. Error bars indicate the standard deviations of three biological replicates. The expression levels of all the six selected genes were relatively high and significantly induced by BPH infestation (24 h) in the resistant varieties RH, ‘PTB33’, ‘R644’, ‘B5’ and ‘IR54751’, but were relatively low and barely induced by BPH infestation in the susceptible varieties TN1, ‘Bai56’, ‘Nipponbare’, ‘Zhenshan 97B’ and 9311. (JPG 2037 kb) [file 12870_2017_1005_MOESM7_ESM.jpg]
