# Supplementary material for: Gene expression and plant hormone levels in two contrasting rice genotypes responding to brown planthopper infestation
Source: BMC Plant Biol. 2017 Feb 28;17:57. doi: 10.1186/s12870-017-1005-7 (PMC5331639; doi:10.1186/s12870-017-1005-7)
Supplement: Additional file 8: — All of the primers used in this study. (DOCX 22 kb) [file 12870_2017_1005_MOESM8_ESM.docx]

Supplementary table 1 All the primer were used in this study.

| Primer name | primer sequence | primer describtion |
| --- | --- | --- |
| 01g03310-F | GCTACTTCCATCCTGTTCT | qRT-PCR test |
| 01g03310-R | GTATGGTGTTGGTGGTGG |  |
| 01g03310-G-F | CTCAAAGCAAGCAAGACGTCC | genomic sequence |
| 01g03310-G-R | CTCTCTGCTTGTTATACAGAG |  |
| 01g03310-P-F | GCTGCAACAATAGCGTGG | promotor sequence |
| 01g03310-P-R | GGACGTCTTGCTTGCTTTGAG |  |
| 01g03320-F | AACAAACAGGCGAAATCC | qRT-PCR test |
| 01g03320-R | CAGACGAAGACGACATCT |  |
| 01g03320-G-F | CCTAGCTAGCTAATAAGCTCC | genomic sequence |
| 01g03320-G-R | GCTTGATATAGCTAGCTATAG |  |
| 01g03320-P-F | GTGAGTGGACTGATTCATGG | promotor sequence |
| 01g03320-P-R | CTTTGCTTGGAGCCTTGGAG |  |
| 06g49190-F | CCATCCTACCGCAGCTAC | qRT-PCR test |
| 06g49190-R | TTAGCAGTTGGCCTTGAC |  |
| 06g49190-G-F | CACACTGACACACAGTCACACAC | genomic sequence |
| 06g49190-G-R | CAACAAGATTCCCATTGAAGAG |  |
| 06g49190-P-F | ATCGACATCCCCTTCTCCTT | promotor sequence |
| 06g49190-P-R | TCGACTTGGCTCTTGGTTTT |  |
| 08g07100-F | ACCACTATGCTTCCACAA | qRT-PCR test |
| 08g07100-R | CCACGAATCTTCTATCAACTC |  |
| 08g07100-G-F | CACTCACCAGATAGTTGCAGA | genomic sequence |
| 08g07100-G-R | CATGATCAGCAGCCACCATC |  |
| 08g07100-P-F | GACAGTATAGAGTGACACAAC | promotor sequence |
| 08g07100-P-R | GTAGACACTCACCAGATAG |  |

| 11g06570-F | GACATTGACCACACCTTG | qRT-PCR test |
| --- | --- | --- |
| 11g06570-R | CATCGCTACTACCGCTAT |  |
| 12g14440-F | TGGCAACTGGTGTTATCT | qRT-PCR test |
| 12g14440-R | GTGATGTCTTGAACTGTCC |  |
| 12g14440-G-F | CTCTGCCAGCAGTAGCTCTCAGCT | genomic sequence |
| 12g14440-G-R | CACACGTGAAACTCAAACA |  |
| 12g14440-P-F | CTCCAGGACAACAAGCATTC | promotor sequence |
| 12g14440-P-R | CTGAGAGCTACTGCTGGCAG |  |
| UBI-F | GTTCGCCCAGTTGACATCTC | reference gene for qRT-PCR |
| UBI-R | CAGATTGTTGAGGTTAGTATTGC |  |
